# Effect of DPP-4i inhibitors on renal function in patients with type 2 diabetes mellitus: a systematic review and meta-analysis of randomized controlled trials

**DOI:** 10.1186/s12944-024-02132-x

**Published:** 2024-05-25

**Authors:** Yong Gong, Xueyan Bai, Donglei Zhang, Xingsheng Yang, Zheng Qin, Yu Yang, Yilun Zhou, Jie Meng, Xin Liu

**Affiliations:** 1 Department of Nephrology, Beijing Tiantan Hospital, Capital Medical University, Beijing, China; 2 Department of Hemotology, Beijing Tiantan Hospital, Capital Medical University, Beijing, China; 3 Department of Hemotology, Zhongnan Hospital, Wuhan University, Wuhan, Hubei China; 4 Department of Cardiology, Beijing Tiantan Hospital, Capital Medical University, Beijing, China; 5 Department of Pathology, Beijing TongRen Hospital, Capital Medical University, Beijing, China; 6 Department of Pharmacy, Beijing Tiantan Hospital, Capital Medical University, Beijing, China

**Keywords:** Dipeptidyl peptidase-4 inhibitors, Estimated glomerular filtration rate, Albumin-to-creatinine ratio, Type 2 diabetes mellitus

## Abstract

**Aims:**

About 20–40% patients with type 2 diabetes mellitus (T2DM) had an increased risk of developing diabetic nephropathy (DN). Dipeptidyl peptidase-4 inhibitors (DPP-4i) were recommended for treatment of T2DM, while the impact of DPP-4i on renal function remained unclear. This study aimed to explore the effect of DPP-4i on renal parameter of estimated glomerular filtration rate (eGFR) and albumin-to-creatinine ratio (ACR) in T2DM.

**Methods:**

A systematic search was performed across PubMed, Embase and Cochrane Library. A fixed or random-effects model was used for quantitative synthesis according to the heterogeneity, which was assessed with I^2^ index. Sensitivity analysis and publication bias were performed with standard methods, respectively.

**Results:**

A total of 17 randomized controlled trials were identified. Administration of DPP-4i produced no significant effect on eGFR (WMD, -0.92 mL/min/1.73m^2^, 95% CI, -2.04 to 0.19) in diabetic condition. DPP-4i produced a favorable effect on attenuating ACR (WMD, -2.76 mg/g, 95% CI, -5.23 to -0.29) in patients with T2DM. The pooled estimate was stable based on the sensitivity test. No publication bias was observed according to Begg’s and Egger’s tests.

**Conclusions:**

Treatment with DPP-4i preserved the renal parameter of eGFR in diabetic condition. Available evidences suggested that administration of DPP-4i produced a favorable effect on attenuating ACR in patients with T2DM.

**International Prospective Register for Systematic Review (PROSPERO) number:**

CRD.42020144642.

**Supplementary Information:**

The online version contains supplementary material available at 10.1186/s12944-024-02132-x.

## Introduction

The number of patients with type 2 diabetes mellitus (T2DM) was increasing annually across the world. An increased morbidity or mortality partially stem from macrovascular and/or microvascular complications occurred during T2DM progression. Diabetic nephropathy (DN), one common microvascular complication, was characterized as a marked decrease of estimated glomerular filtration rate (eGFR) and/or a persistent increase of albuminuria [1]. Evidence suggested that 20–40% of patients developed microvascular complications of DN in diabetic condition [2]. A chronic exposure to hyperglycaemia led to progressive impairment of the renal microvasculature [3]. Therapeutic strategies should not modulate glycaemic balance alone, while other measures including an attenuation of blood pressure and/or preserving renal function should also be performed in diabetic context [4].

Traditional antidiabetic agents mainly focused on glucose control in treatment of T2DM. Dipeptidyl peptidase-4 inhibitors (DPP-4i) were developed as noninsulin hypoglycaemic agents since 2006, and these agents were orally administered in clinical practice. Preclinical study demonstrated that DPP-4 was expressed in the kidney, and increased DPP-4 activity was positively correlated with levels of creatinine and proteinuria [5]. Inhibition of DPP-4 effectively improved renal outcomes by decreasing tubular and glomerular proteinuria in diabetic setting [6]. Evidence indicated that DPP-4i potentially attenuated renal biomarkers for tubular injury in patients with diabetic kidney disease (DKD) [7]. In contrast, some studies yielded different estimates on renal parameters during treatment with DPP-4i. A long-term treatment with linagliptin produced no significant effect on eGFR compared to placebo (-0.8 vs. -2.2 mL/min/1.73 m^2^) in diabetic participants with renal impairment [8]. Similarly, administration of linagliptin did not significantly modulate albuminuria in diabetic individuals with renal dysfunction [9]. However, a pooled analysis demonstrated that treatment with DPP-4i significantly reduced eGFR (-1.11 mL/min/1.73 m^2^; 95% CI, -1.78 to -0.44; *P* = 0.001) in patients with T2DM [10]. It was an important issue to explore the extent to which DPP-4i modulated renal parameters in patients with T2DM. Therefore, this study was performed to evaluate an impact of DPP-4i on eGFR and albumin-to-creatinine ratio (ACR) in patients with T2DM.

## Methods

### Search strategy

This study was designed based on the Preferred Reporting Items for Systematic Reviews and Meta-Analyses (PRISMA) statement [11]. PubMed, Embase and Cochrane Library were searched for trials published before April 30, 2024. Relevant items included (“dipeptidyl peptidase-4 inhibitors” OR “sitagliptin” OR “vildagliptin” OR “teneligliptin” OR “saxagliptin” OR “linagliptin” OR “alogliptin”) AND (“type 2 diabetes” OR “type 2 diabetes mellitus” OR “T2DM”) AND (randomized controlled trials).

### Study selection

Two reviewers screened databases independently and searched the reference lists for eligible articles manually. Randomized controlled trials (RCTs) evaluating the impact of DPP-4i on eGFR and/or ACR were selected. Inclusion criteria were established as follows: (i) an effect of DPP-4i on eGFR or ACR was studied; (ii) relative information on renal parameter was recorded at baseline and follow-up, or a change was indicated directly; and (iii) patients were diagnosed with T2DM. The exclusion criteria were listed as follows: (i) non-human studies; (ii) lack of records on eGFR or ACR; and (iii) meetings, abstracts or reviews.

### Data extraction

Detailed records were extracted into the table, including (i) first author; (ii) publication year; (iii) trial location; (iv) number of participants in DPP-4i and control groups; (v) age and body mass index (BMI); (vi) follow-up and diabetes duration; (vii) HbA1c% at baseline; and (viii) eGFR and ACR at baseline. Studies with multiple follow-ups were extracted as the longest duration.

### Quality assessment

Quality of RCTs was evaluated based on the Cochrane criteria [14]. Related items included random sequence generation, allocation concealment, blinding of participants, personnel, outcome assessment, incomplete outcome data, selective outcome reporting, and other potential sources of bias. A judgement of ‘yes’ indicated a low risk of bias, while a judgement of ‘no’ indicated a high risk of bias. A judgement of ‘unclear’ indicated an unknown or unclear risk of bias.

### Quantitative data synthesis

A pooled calculation was performed on the renal parameter of eGFR or ACR. Weighted mean difference (WMD) and 95% confidence interval (CI) were calculated for changes of eGFR and ACR. A fixed- or random-effects model was used according to the heterogeneity, which was quantified by the index of I^2^. Sensitivity test was used to examine the influence of individual study on an overall estimate. In case of possible important heterogeneity, subgroup analysis was accordingly performed on related parameters. Publication bias was also examined by Begg’s and Egger’s tests if there were at least five studies reporting changes of eGFR or ACR. All these analysis were performed by using Review Manager (5.3) and STATA (12.0) software.

## Results

### Characteristics of the included studies

**Fig. 1:**
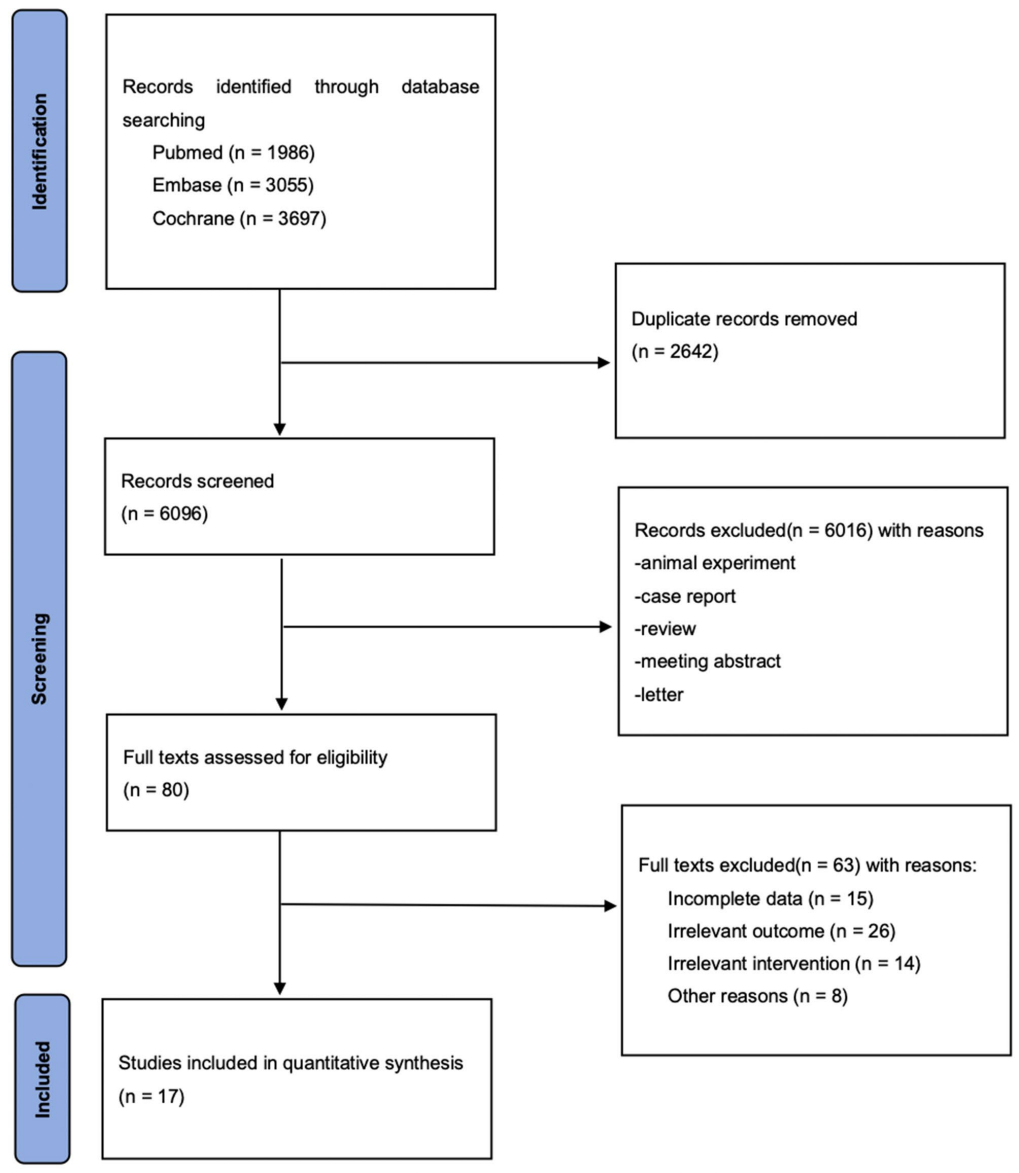
PRISMA flow chart for study selection

The literature search produced 8,738 records, and 17 publications (19 studies) met an inclusion criteria (Fig. 1). In addition, 17 studies reported the change of eGFR, while 11 studies reported the change of ACR during DPP-4i treatment. Fourteen studies lasted less than 1 year (ranging from 1 to 6 months), and three studies lasted longer than 1 year (ranging from 13 to 26 months). Two studies had a sample size of larger than 100, respectively. Characteristics of eligible were detailed illustrated (Table 1).

**Table 1:** Demographic characteristics of the studies included

Study/year	Location	Treatment arm (*n*)	follow-up (weeks)	Duration of diabetes (years)	BMI (kg/m^2^)	HbA1c (%)	eGFR (mL/min/1.73m^2^)	ACR (mg/g)
Narimani, 2021 [41]	Iran	sita(50 mg):43 pla(50 mg):41	12	14.4 ± 3.6 14.9 ± 2.8	28.0 ± 3.6 28.8 ± 4.7	7.9 ± 0.4 7.8 ± 0.3	73.3 ± 10.7 72.7 ± 7.7	314.4 ± 414.6 298.7 ± 427.4
Cosenso-Martin, 2018 [42]	Brazil	vild(100 mg):24 glib(100 mg):24	12	6.9 ± 5.6 5.92 ± 4.0	31.5 ± 3.3 30.0 ± 3.5	8.3 ± 1.0 7.9 ± 0.9	86.2 ± 16.0 91.2 ± 17.5	25.7 ± 19.1 22.1 ± 20.1
Ott, 2016 [43]	Germany	lina(5 mg):30 pla(5 mg):32	4	3.8 ± 3.3 5.1 ± 3.0	29.6 ± 4.0 29.8 ± 4.8	7.0 ± 0.7 6.8 ± 0.8	140.0 ± 14.0 141.0 ± 15.0	NS
Suzuki, 2014 [44]	Japan	sita(50 mg):16 lira(0.9 mg):24	24	1.9 ± 2.3 2.4 ± 2.8	26.3 ± 7.2 28.2 ± 7.2	9.1 ± 1.6 9.8 ± 2.2	73.7 ± 12.6 73.2 ± 13.4	23.4 ± 31.0 40.2 ± 62.0
Mori, 2014 [45]	Japan	sita(50 mg):42 con:38	24	10.0 ± 6.7 8.8 ± 6.5	25.2 ± 4.2 25.3 ± 4.1	7.0 ± 0.7 6.9 ± 0.7	77.1 ± 18.9 75.5 ± 28.1	68.9 ± 133.4 61.4 ± 154.3
Dei Cas, 2017 [46]	Italy	vild(100 mg):40 glib(2.5 ~ 5 mg):24	48	7.3 ± 5.2 5.3 ± 6.7	29.6 ± 4.5 29.5 ± 6.4	7.7 ± 0.4 7.8 ± 0.4	96.1 ± 11.8 96.0 ± 14.5	NS
Takihata, 2013 [47]	Japan	sita(50 mg):58 piog(15 mg):57	24	NS	24.6 ± 3.3 25.8 ± 4.8	7.5 ± 0.7 7.4 ± 0.6	87.6 ± 17.5 88.1 ± 19.6	80.8 ± 185.0 100.5 ± 248.0
Lovshin, 2017 [48]	Canada	sita(100 mg):16 pla(100 mg):16	4	6.3 ± 5.2 9.3 ± 6.3	31.7 ± 5.5 30.2 ± 7.0	7.2 ± 0.8 7.3 ± 0.8	94.1 ± 7.2 94.2 ± 11.4	NS
Tonneijck, 2016 [15]	Netherlands	sita(100 mg):19 pla(100 mg):17 Lira(1.8 mg):19	12	7.3 ± 5.9 8.3 ± 5.2 8.0 ± 6.7	31.5 ± 5.7 30.4 ± 1.9 32.9 ± 3.7	7.1 ± 0.5 7.5 ± 0.7 7.4 ± 0.7	92.0 ± 13.0 90.0 ± 15.0 93.0 ± 12.0	16.6 ± 23.6 19.0 ± 28.9 8.4 ± 5.3
Zografou, 2015 [49]	Greece	vild(100 mg) + met(1700 mg):32 met(1700 mg):32	24	NS	31.6 ± 4.6 32.2 ± 5.9	8.1 ± 0.8 8.0 ± 0.8	122.0 ± 30.2 123.1 ± 35.2	26.2 ± 40.1 18.4 ± 19.3
Hayashi, 2017 [50]	Japan	sita(50 mg):40 dapa(5 mg):40	12	NS	NS	7.5 ± 1.6 7.6 ± 1.1	83.5 ± 22.7 86.2 ± 18.4	NS
Mita, 2018 [51]	Japan	lina(5 mg):21 met(500-2250 mg):20	24	3.4 ± 5.9 3.3 ± 4.1	25.7 ± 4.5 26.3 ± 4.9	7.1 ± 0.7 7.5 ± 1.5	76.7 ± 17.2 92.0 ± 21.4	21.3 ± 28.3 19.5 ± 21.0
Nakamura, 2014 [52]	Japan	sita(50 mg):24 vog(0.6 mg):31	12	4.8 ± 3.4 3.5 ± 3.7	27.8 ± 3.5 25.7 ± 4.3	7.0 ± 0.6 6.9 ± 0.4	66.8 ± 20.8 63.6 ± 20.8	NS
Oe, 2015 [53]	Japan	sita(50 mg):38 vog(0.6 mg):39	24	4.0 ± 356.0 3.2 ± 331.6	27.7 ± 4.1 25.7 ± 4.3	7.1 ± 0.7 6.9 ± 0.5	75.0 ± 22.0 71.0 ± 15.0	NS
Mita, 2015 [54]	Japan	alog(25 mg):150 con:153	104	9.7 ± 7.4 9.1 ± 8.1	24.6 ± 4.3 24.9 ± 3.7	7.3 ± 0.8 7.2 ± 0.8	78.0 ± 20.0 77.0 ± 18.0	25.3 ± 34.4 23.2 ± 29.0
Yamada, 2017 [55]	Japan	sita(25–100 mg):55 con(100 mg):60	96	NS	25.9 ± 3.3 24.8 ± 3.9	7.0 ± 0.6 6.9 ± 0.5	66.6 ± 15.9 67.3 ± 18.4	NS
Roden, 2015 [56]	Germany	sita(100 mg):136 empa(10 mg or 25 mg):143 pla(100 mg):119	76	NS	28.2 ± 5.2 28.2 ± 5.5 28.7 ± 6.2	7.8 ± 0.8 7.9 ± 0.8 7.9 ± 0.8	87.6 ± 17.3 87.5 ± 18.3 86.8 ± 17.9	NS

Values are expressed as mean ± SD. Abbreviations: n, number of participants per group; sita, sitagliptin; vild, vildagliptin; alog, alogliptin; empa, empagliflozin; pla, placebo; piog, pioglitazone; con, conventional treatment; met, metformin; dapa, dapagliflozin; lira, liraglutide; vog, voglibose; lina, linagliptin; glib, glibenclamide; NS, not stated

### Quality evaluation

**Fig. 2:**
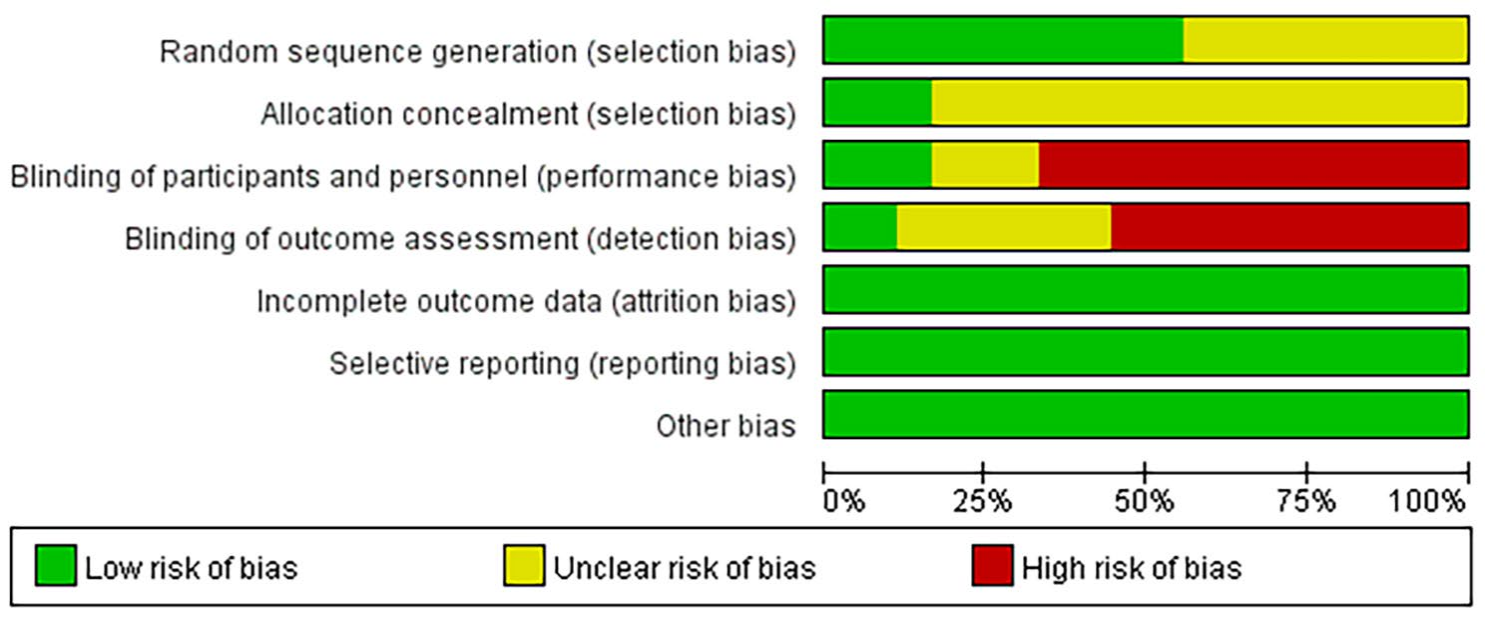
Risk of bias assessment in the studies identified for meta-analysis

Study quality was objectively evaluated by two reviewers with Cochrane criteria(Fig. 2). All the studies were randomly designed, and three studies provided sufficient data about allocation concealment. Ten studies had detection bias on the basis of blinding of outcome assessment. Additionally, thirteen trials had performance bias as blinding methods were not implemented.

### Effect of DPP-4i on eGFR in T2DM

**Fig. 3:**
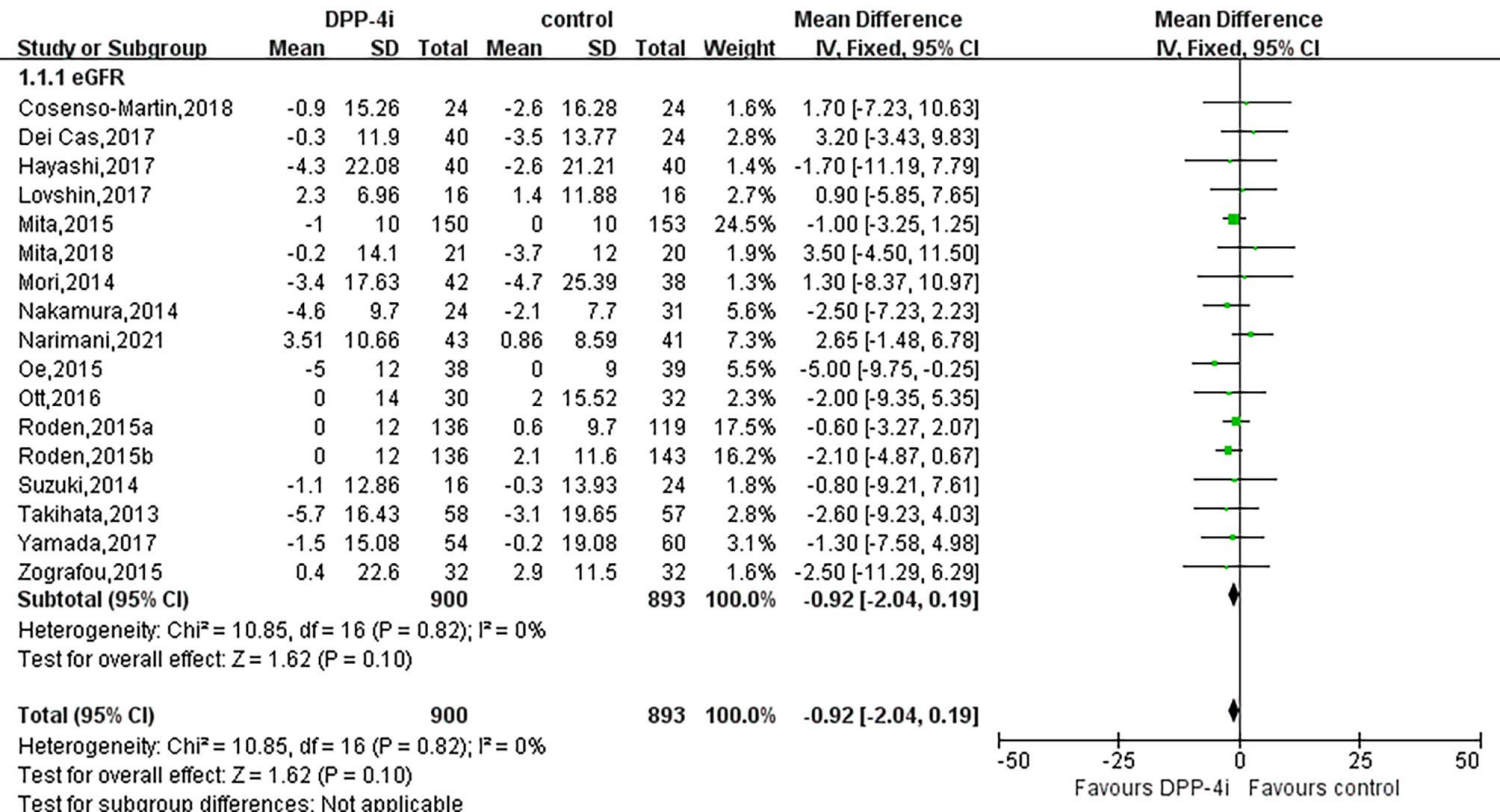
Forest plot for the impact of DDP-4i versus placebo or active comparators on eGFR

A pooled estimate suggested that administration of DPP-4i preserved eGFR (WMD, -0.92 mL/min/1.73 m^2^, 95% CI, -2.04 to 0.19, I^2^ = 0%, *P* = 0.10) in patients with T2DM (Fig. 3). Subgroup analysis indicated that HbA1c at baseline, lengths of follow-up, BMI, comparator type and dosage did not influence the effect of DPP4i on the eGFR. In addition, no significant differences were observed in subgroups of DPP-4i alone, combined with other antidiabetic agents or inhibitors of renin-angiotensin-aldosterone system (RAASi) (Table 2).

**Table 2:** Subgroups analysis on the correlation of DDP-4i with eGFR and ACR in T2DM

Variables	Subgroups	RCTs (*n*)	WMD, 95% CI	I^2^	*P*	*P* value for interaction
eGFR	Placebo	6	-0.7, -2.3 to 1.0	0%	0.4	0.54
	Active agents	11	-1.2, -2.7 to 0.4	0%	0.1	
	Follow-up < 24weeks	6	0.2, -2.3 to 2.6	0%	0.9	0.27
	Follow-up ≥ 24weeks	11	-1.2, -2.5 to 0.04	0%	0.1	
	DPP-4i monotherapy	7	-0.5, -2.1 to 1.1	0%	0.1	0.65
	Combination therapies	10	-1.3, -2.9 to 0.2	0%	0.6	
	HbA1c ≤ 7.5%	9	-1.4, -3.0 to 0.1	0%	0.1	0.90
	HbA1c > 7.5%	8	-0.4, -2.0 to 1.1	0%	0.6	
	BMI < 30	13	-1.0, -2.1 to 0.2	0%	0.1	0.64
	BMI ≥ 30	3	0.2, -4.4 to 4.8	0%	0.9	
	DPP-4i monotherapy	6	-1.2, -2.9 to 0.5	0%	0.2	0.39
	Coadministration with RAASi	11	-0.7, -2.2 to 0.7	0%	0.3	
	Sitagliptin at dose of 50 mg	7	-1.2, -3.4 to 1.0	8%	0.3	0.52
	Sitagliptin at dose of 100 mg	4	-1.2, -2.9 to 0.6	0%	0.2	
ACR	Placebo	3	-11.5, -23.5 to 0.6	0%	0.1	0.28
	Active agents	8	-2.4, -4.9 to 0.1	0%	0.1	
	Follow-up < 24weeks	4	-5.6, -12.2 to 1.1	0%	0.1	0.65
	Follow-up ≥ 24weeks	7	-2.3, -5.0 to 0.3	0%	0.1	
	DPP-4i monotherapy	4	-5.9, -15.0 to 3.2	25%	0.2	0.53
	Combination therapies	7	-2.5, -5.1 to 0.1	0%	0.1	
	HbA1c ≤ 7.5%*	7	-2.6, -5.2 to -0.01	0%	0.0	0.92
	HbA1c > 7.5%	4	-4.2, -12.1 to 3.7	0%	0.3	
	BMI < 30	7	-2.1, -4.8 to 0.6	0%	0.1	0.58
	BMI ≥ 30*	4	-6.2, -12.2 to -0.1	0%	0.0	
	DPP-4i monotherapy	2	-9.1, -22.9 to 4.8	0%	0.2	0.48
	Coadministration with RAASi*	9	-2.6, -5.1 to -0.1	0%	0.1	

* Pooled analysis was significantly demonstrated in relative group

### Effect of DPP-4i on ACR in T2DM

**Fig. 4:**
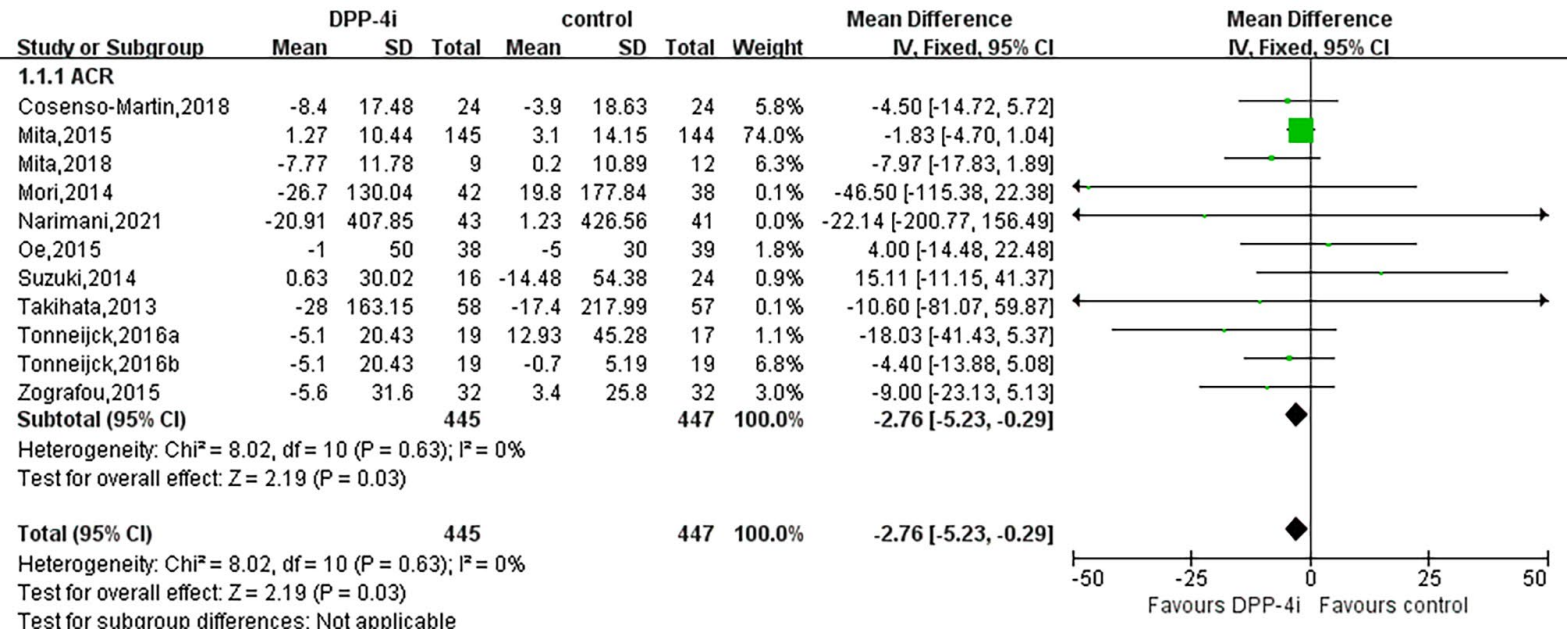
Forest plot for the impact of DDP-4i versus placebo or active comparators on ACR

Administration of DPP-4i produced a significant effect on reducing ACR (WMD, -2.76 mg/g, 95% CI, -5.23 to -0.29, I^2^ = 0%, *P* = 0.03) in T2DM (Fig. 4). In addition, DPP-4i significantly reduced ACR in subgroups of HbA1c ≤ 7.5, BMI ≥ 30 kg/m^2^ and coadministration of RAASi. However, no significant effects were indicated in subgroups of BMI, comparator type or coadministration with other antidiabetic agents during DPP4i treatment (Table 2).

### Evaluation of publication bias

The pooled estimates on eGFR and ACR were stable according to the leave-one-out sensitivity test (Supplementary Figs. 1–2). This result proved that a significant difference was an overall effect of all the identified studies. No publication bias was observed on the association of DDP4i with eGFR or ACR according to Begg’s test (eGFR, *P* = 0.48, ACR, *P* = 1.00) or Egger’s test (eGFR, *P* = 0.478, ACR, *P* = 0.217) (Supplementary Figs. 3–4). In addition, no significant interactions were detected on the pooled estimates of eGFR or ACR across subgroup analysis (Table 2).

## Discussion

DPP-4i were commonly recommended for treatment of patients with or without DN. Pooled analysis demonstrated that DPP-4i preserved renal function of eGFR in patients with T2DM. This finding was consistent with that of previous study in which DPP-4i were safely administered in diabetic patients with or without chronic kidney disease (CKD). Administration of sitagliptin resulted in no significant change of eGFR as that of glipizide in diabetic patients with CKD [12]. Additionally, outcomes from the Trial Evaluating Cardiovascular Outcomes with Sitagliptin (TECOS) demonstrated that sitagliptin did not significantly modulate eGFR after a long-term treatment [13]. A retrospective analysis also uncovered that teneligliptin could be safely used at an early stage in diabetic patients with DKD [14]. In addition, sitagliptin did not significantly modulate eGFR (-6 mL/min/1.73 m^2^, 95% CI, -14 to 3) in overweight patients with T2DM [15]. Similarly, a nonsignificant change of eGFR was observed in subgroup analysis on BMI. Pooled estimates might stem from a lack of significant renal haemodynamic changes during DPP-4i treatment. Different hyperfiltration ranges might also participate in attenuating eGFR in T2DM [16].

The preserved effect of DPP-4i on eGFR was consistent with outcomes of SAVOR-TIMI 53 trial. Saxagliptin did not significantly modulate eGFR while showing a beneficial effect on ACR in T2DM [17]. The pooled analysis also revealed that DPP-4i favorably reduced ACR in patients with T2DM. A preclinical study showed that DPP-4i reduced ACR and slowed the progression of renal impairment independent of blood pressure [18]. Evidence showed that saxagliptin and vildagliptin significantly reduced albuminuria, respectively, in diabetic patients with hypertension. Saxagliptin might present a stronger effect on reducing albuminuria compared to vildagliptin, an action independent of glycaemic control [19]. In fact, administration of saxagliptin ameliorated microalbuminuria in patients with or without renal impairment [20]. These results indicated that DPP-4i might produce an effect on ACR in a direct pathway. Most DPP-4i were predominantly excreted by the kidneys, except for linagliptin. A pooled analysis demonstrated that linagliptin significantly reduced ACR in patients receiving treatment of RAASi [21]. Subgroup analysis revealed that DPP-4i significantly reduced ACR in subgroup of HbA1c < 7.5. A previous study revealed that no significant correlation of DPP-4i with albuminuria was found in patients with different levels of HbA1c [20]. This might come from multiple parameters applied by different teams, namely, ACR and albuminuria alone. Correlation analysis also indicated that changes of ACR were associated with eGFR and systolic blood pressure in sitagliptin-treated participants [22]. This analysis suggested that an impact of DPP-4i on ACR partially dependent on eGFR at baseline. A significant effect of DPP-4i on ACR was also observed in patients with BMI > 30 kg/m^2^, while the underlying mechanism remained unclear in patients with T2DM.

The potential mechanism by which DPP-4i attenuated renal function might involve multiple pathways. First, DPP-4i increased the levels of glucagon-like peptide-1 (GLP-1), thereby inhibiting glomerular hyperfiltration [23]. Second, inflammation played a key role in the progression of CKD, and DPP-4i produced an anti-inflammatory effect by targeting toll-like receptor 4 (TLR4) in diabetic model [24]. Third, oxidative stress participated in the occurrence of renal impairment. Vildagliptin alleviated the process of renal sclerosis by inhibiting p22phox in diabetic condition [25]. DPP4i also significantly reduced an accumulation of reactive oxygen species (ROS) and promoted the activation of superoxide dismutase (SOD). DPP-4i reduced oxidative stress through modulating haem oxygenase-1 (HO-1) and NF-E2-related factor 2 (Nrf2) [26]. Fourth, kidney fibrosis was recognized as a final step in progression of CKD, which was ameliorated by an inhibition of endothelial-to mesenchymal transition (EndMT) during DPP-4i treatment [27]. In addition, DPP-4i produced a vasodilating effect on vessels by inducing a release of endothelial nitric oxide synthase (eNOS) [28]. Finally, DPP-4i improved pancreatic β-cell function in both fasting and postprandial states in patients with T2DM, which potentially presented vasodilatory effects on renal system [29].

In addition to DPP-4i, other antidiabetic agents had been reported to exert multiple effects on renal function in T2DM. Incretin-based GLP-1 receptor agonists (GLP-1RA) could improve renal function by presenting an antioxidant and/or anti-atherosclerotic effect in diabetic condition. Evidence demonstrated that weight reduction also contributed to a decrease of albuminuria during semaglutide treatment [30]. Administration of sodium–glucose cotransporter 2 inhibitors (SGLT2i) was reported to show a transient reduction of eGFR and proteinuria in diabetic patients [31]. The reduction of glomerular filtration might result from an effect of renal adenosine under hyperglycaemic conditions [32]. Metformin was proved to improve renal function by slowing the progression of kidney fibrosis. Preclinical evidence suggested that metformin targeted the AMPK signalling pathway, thus contributing to the normalization of kidney structure [33]. Pioglitazone, a peroxisome proliferator-activated receptor γ (PPAR-γ) agonist, was also found to modulate the progression of renal fibrosis and ameliorate DN in diabetic model [34]. Pioglitazone showed a reno-protective effect by attenuating mitochondrial function and stabilizing membrane potential [35]. Similarly, glibenclamide stabilized kidney structure by downregulating an expression of inflammatory markers. This action was accompanied with an alleviation of inflammatory cell infiltration in the kidney [36]. In the present study, DPP-4i did not demonstrate a stronger effect on renal parameters compared to other antidiabetic agents. The composite impact of multiple agents might ultimately surpass the effects of DPP-4i on renal parameters in diabetic participants. Head-to-head studies comparing DPP-4i with other antidiabetic agents should be designed to evaluate the effect on eGFR.

Finally, given the protective effect of RAASi on DN, it was important to determine whether DPP-4i showed a synergistic effect on renal function with RAASi. Angiotensin II (Ang II) downregulated the expression of megalin by activating DPP-4 in the proximal tubules, thereby resulting in an impairment of renal function. Inhibition of DPP-4 upregulated the expression of megalin in an Ang II-mediated way, thus decreasing the phosphorylation of extracellular regulated kinase (ERK) [37]. Linagliptin marked decreased glycosylated haemoglobin levels and preserved renal function when added to a conventional dose of RAASi in DN [38]. A pooled estimate demonstrated that coadministration of DPP-4i with RAASi produced a favorable effect on reducing ACR in T2DM. Evidence uncovered that an addition of DPP-4i to a maximal dose of RAASi markedly reduced ACR in patients with renal dysfunction [39]. This suggested that coadministration of DPP-4i with RAASi produced a synergistic effect on improving renal function in diabetic patients with renal impairment. In addition, a previous study showed that sitagliptin potentially targeted the sympathetic nervous system, thus weakening the hypotensive effect of angiotensin-converting enzyme inhibitors (ACEI) in patients with metabolic syndrome [40]. Therefore, essential measures should be performed to monitor blood pressure when patients received a maximal dose of RAASi during treatment with DPP-4i.

### Strengths

This meta-analysis had some strengths to be stated. This meta-analysis firstly combined evidence on changes of eGFR and ACR during DPP-4i treatment. Pooled results suggested that DPP-4i potentially produced a favorable effect in patients with DN. In addition, subgroup analysis was performed to explore the influence of related parameters on renal function.

### Limitations

It should be noted that this study had some limitations. Firstly, included studies had relatively small sample sizes, and a few number of trials were identified. Secondly, the identified trials showed differences in characteristics of participants, eGFR or ACR at baseline, and dosage of DPP-4i. Variations of these parameters might present an impact on an overall estimate. Thirdly, only publications in related databases were included, which also produce an inevitable publication bias.

## Conclusions

Administration of DPP-4i potentially reduced ACR and prevented the decline of eGFR in T2DM. These results suggested that diabetic participants with or without albuminuria potentially benefit more from DPP-4i treatment in clinical practice.

### Electronic supplementary material

Below is the link to the electronic supplementary material.

Supplementary Material 1

Supplementary Material 2

Supplementary Material 3

Supplementary Material 4

## Data Availability

No datasets were generated or analysed during the current study.

## Abbreviations

T2DM: Type 2 diabetes mellitus
DN: Diabetic nephropathy
DPP-4i: Dipeptidyl peptidase-4 inhibitors
eGFR: Estimated glomerular filtration rate
ACR: Albumin-to-creatinine ratio
DKD: Diabetic kidney disease
PRISMA: Preferred Reporting Items for Systematic Reviews and Meta-Analyses
BMI: Body mass index
WMD: Weighted mean difference
CI: Confidence interval
CKD: Chronic kidney disease
TECOS: Trial Evaluating Cardiovascular Outcomes with Sitagliptin
RAASi: Inhibitors of renin-angiotensin-aldosterone system
TLR4: Toll-like receptor 4
ROS: Reactive oxygen species
SOD: Superoxide dismutase
HO-1: Heme oxygenase-1
Nrf2: NF-E2-related factor 2
EndMT: Endothelial-to mesenchymal transition
eNOS: Endothelial nitric oxide synthase
GLP-1: Glucagon-like peptide-1
GLP-1RA: GLP-1 receptor agonists
SGLT2i: Sodium–glucose cotransporter 2 inhibitors
AMPK: AMP-activated protein kinase
PPAR-γ: Proliferator-activated receptorγ
And II: Angiotensin II
ERK: Extracellular regulated kinase
ACEI: Angiotensin-converting enzyme inhibitors
