# Supplementary figures and images for: Effect of DPP-4i inhibitors on renal function in patients with type 2 diabetes mellitus: a systematic review and meta-analysis of randomized controlled trials
Source: Lipids Health Dis. 2024 May 25;23:157. doi: 10.1186/s12944-024-02132-x (PMC11128128; doi:10.1186/s12944-024-02132-x)

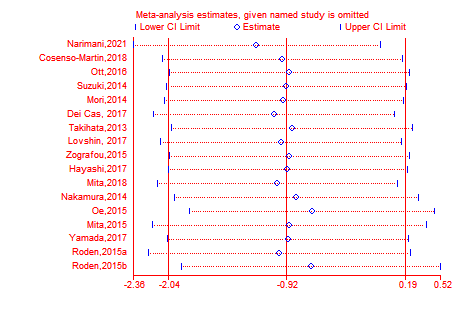

Supplement: Supplementary file 1 — Supplementary Material 1 [file 12944_2024_2132_MOESM1_ESM.png]

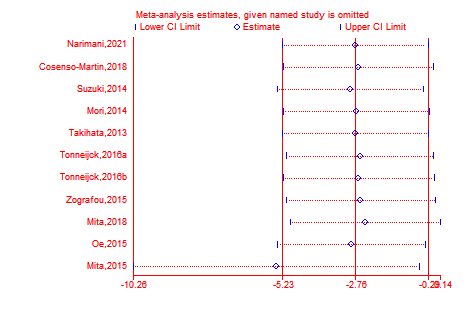

Supplement: Supplementary file 2 — Supplementary Material 2 [file 12944_2024_2132_MOESM2_ESM.png]

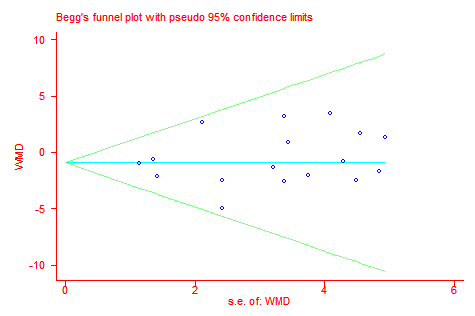

Supplement: Supplementary file 3 — Supplementary Material 3 [file 12944_2024_2132_MOESM3_ESM.png]

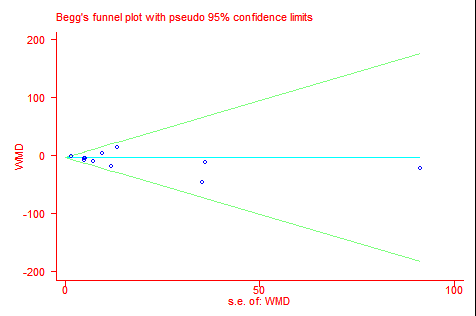

Supplement: Supplementary file 4 — Supplementary Material 4 [file 12944_2024_2132_MOESM4_ESM.png]
